# Understanding the expectations, positions and ambitions of LMICs during pandemic treaty negotiations, and the factors contributing to them

**DOI:** 10.1371/journal.pgph.0003851

**Published:** 2025-03-12

**Authors:** Greta Cranston

**Affiliations:** 1 Sydney School of Public Health, The University of Sydney, Sydney, New South Wales, Australia; Tony Blair Institute for Global Change, UNITED KINGDOM OF GREAT BRITAIN AND NORTHERN IRELAND

## Abstract

Global South countries account for two-thirds of WHO Member States and are a crucial voice in negotiating the ‘pandemic treaty’, which Member States agreed was necessary if the world was to avoid a repeat of the significant inequity that resulted during COVID-19. The negotiation of a pandemic treaty presents an opportunity to recalibrate global health systems and processes for pandemic prevention, preparedness and response. However, for this to eventuate through global solidarity, as many Global South countries have said they expect, then concessions by developed states on issues that they have long protected must occur. It remains to be seen whether the negotiations amount to a rebalancing of power and resources to substantially improve our capacity and capability to address global health threats. Further, while these issues have often been viewed through a dualistic lens between developing and developed countries, these negotiations also reflect the diversity of Global South Member States, some of which are strong voices in, and for, coalitions. Drawing on content analysis techniques, this research describes how Global South countries articulate their expectations, positions and ambitions during treaty negotiations in the lead up to the World Health Assembly in 2024 and discusses exogenous and endogenous factors that contribute to these positions. The analysis found that the pursuit of equity is galvanising for Global South countries, as are their ambitions for the multilateral system not to drive further inequity through the asymmetry of power and influence exercised by developed states. Well-coordinated collective positions from Global South countries in negotiations to date have resulted in traction on contested issues such as technology transfer, production capability, and access and benefits. Additionally, some Global South countries have also used these negotiations to pursue national interests whilst maintaining collective positions in pursuit of equity and global solidarity.

## Introduction

The COVID-19 pandemic highlighted that the entities, systems and processes underpinning the practice of global health security are “fundamentally affected by the distribution of power in the international system” [1] and captured by the “self-interested behaviour of sovereign states”[2]. The unprecedented detrimental impact of COVID-19 was subject to much review, including by the Independent Panel for Pandemic Prevention and Response (referred to herein as ‘The Panel’) [3]. The Panel recommended the principles of global solidarity and a cosmopolitan approach to promote equity and improve pandemic prevention, preparedness and response (PPPR). Cosmopolitanism is defined as the primacy of individuals and their rights within a global citizenry [4,5] and the moral responsibility to protect all people regardless of state borders [6]. In the context of the pandemic treaty to affect equity for global PPPR, cosmopolitanism and global solidarity are contrasted with a realist approach, defined as nation-states prioritising their security and interests above others [7–9]. The Panel’s recommendation for an internationally binding agreement for PPPR to elicit political and multisectoral commitment [3] was adopted in December 2021, when World Health Organization (WHO) Member States agreed to develop “a WHO convention, agreement or other international instrument on pandemic preparedness and response*”* [10] (referred to herein as the ‘pandemic treaty’).

The decision to negotiate a pandemic treaty acknowledges that the realist approach to health security has undermined global PPPR efforts. This was starkly illustrated by the inequity and zero-sum game of ‘vaccine nationalism’ whereby predominantly low-and middle-income countries (LMICs) went without timely access to COVID-19 vaccines resulting in a world divided into ‘haves’ and ‘have nots’. Consequently, the ensuing pandemic treaty negotiations have sharpened the focus on the degree to which inequity is negated, perpetuated or ameliorated by global health entities, systems and processes [11]. As these negotiations continue it remains to be seen what impact the treaty has on affecting equity in principle, and in practice, for global health. Importantly, this includes the degree to which geopolitical and economic factors influence Member State negotiations and their appetite to reduce inequity and broker solutions, including on some of the most contentious aspects of the treaty involving equitable access to countermeasures and associated technologies, knowledge and capabilities [12,13].

As the Intergovernmental Negotiating Body (INB) approached the end of its initial timeline to agree a pandemic treaty, this research analyses INB meeting sessions between November 2023 and March 2024, to hear how LMICS voice their expectations, positions and ambitions during treaty negotiations and their visions for equity. Commentary about the significant, contentious and potentially irreconcilable differences arising out of these negotiations have often been presented as a dualistic tension between the LMICs comprising the Global South with equity central to their objectives, and the wealthy developed states of the Global North, frequently characterised as unwilling to make necessary concessions [14] in the multilateral system for the benefit of all.

The term ‘Global South’ infers a homogenous group that risks veiling the diversity and differences across LMICs and how this may shape and inform their various expectations, positions and ambitions. Illustrating the diversity of LMICs helps to provide further insight into LMIC positions. A system that enables this is the World Bank’s income classification system, which categorises LMICs to broadly “reflect a country’s level of development” [15]. While acknowledging the limitations of this categorisation system, it can suggest differences in economic and political capabilities that may influence the expectations, ambitions and priorities various LMIC Member States seek to prosecute.

As PPPR will continue to be discussed in the multilateral system and LMICs comprise around 65% of WHO’s 194 Member States [16], it is necessary to understand the positions of LMIC Member States in view of how this influences global health diplomacy, PPPR and the continuing pandemic treaty negotiations. Furthermore, these negotiations reveal how some LMICs have prosecuted national objectives without needing to break from their asserted collective positions, which has served to increase LMIC influence in this process.

Adding to the complexity of these negotiations is the concept of equity not being commonly defined nor commonly understood [17]. In the case of global health, including PPPR, operationalising equity suggests “those who have suffered exclusion or marginalization will generally need more, not the same, resources, to address the toll” [18]. If the intention is to repair the fault lines of global health inequity, it is crucial to understand how LMICs envision the operationalisation of equity through the treaty. A good deal hangs on a pandemic treaty that can catalyse political commitment and multilateral reforms to recalibrate the influence and distribution of power and resources in global health. The Panel considers that being better prepared for the next pandemic depends on it.

The period from November 2023 (INB7) through to March 2024 (INB9), originally anticipated as the endpoint of treaty negotiations, provides insight into how LMICs are prosecuting their ‘equity asks’ amidst a backdrop of geostrategic competition, rivalry and conflict [19]. During this time, states accelerated their bargaining [20], detail and exchange [21] and were explicit and assertive in their positions. Drawing upon webcasts of the INB meetings in this period [22–24], this research describes the various expectations, positions and ambitions of LMICs towards the pandemic treaty and discusses the endogenous and exogenous factors contributing to these positions.

## Methods

This descriptive study draws on content analysis [25] and mixed methods techniques to explore the expectations, positions and ambitions of LMICs towards a pandemic treaty, and uses the primary data contained within WHO’s INB webpages [26] including webcast sessions for the negotiation of the proposal for the pandemic treaty [27–29]. Webcasts were viewed for INB7 [22], INB8 [23] and INB9 [24] (these webcasts have no individual URL links as they are embedded in the respective INB meeting webpages, and referenced as such). These INB meetings involved text negotiations where LMIC Member States were assertive and explicit in their expectations, positions and ambitions, as the period between November 2023 through to March 2024 was anticipated to be within the final six-months of treaty negotiations. However, as it became evident the treaty could not be finalised in March 2024, further meetings were scheduled (29 April–10 May and 20–24 May) to determine a way forward [29,30], which are not included in this research.

Two versions of the proposed treaty text were used as the basis for negotiations during the research period: A/INB/7/3 [31] for INB7 and INB8, and A/INB/9/3 [32] for the first session of INB9. The operational components of the proposed treaty are largely contained in the seventeen Articles (4 through to 20) comprising Chapter II, titled: “The world together equitably: achieving equity in, for and through pandemic prevention, preparedness and response” [31,32] that were the focus of the negotiations [33] during the research period. This research uses the article headings of Chapter II as a deductive coding framework [34] for the data analysis to organise and describe how LMICs frame their expectations, positions and ambitions for the operationalisation of equity in the pandemic treaty.

Important to this analysis is the distinction between ‘developing’ and ‘developed’ countries, and the differentiation across developing countries. As these terms are not defined in INB documents nor the Constitution of the World Health Organization [35], the World Bank’s income categorisation of low-income, lower-middle-income and upper-middle-income countries [36] is employed to illustrate the diversity of LMIC Member States. Geographical differentiation is achieved by categorising LMIC Member States by WHO Region (Africa; the Americas; Eastern Mediterranean; Europe; South-East Asia, and Western Pacific) [16].

### Data collection and analysis

Over fifteen hours of INB webcast sessions [37] for INB7, INB8 and INB9 were viewed, and oral statements and interventions made by LMIC Member States were manually transcribed into Microsoft Word. Coding and analysis was conducted, drawing on basic, interpretative and qualitative content analysis techniques [25] and quantitative frequency observations were used to guide the identification of priorities for the qualitative analysis.

#### Quantitative analysis.

The tables below draw on the quantitative observations in the Supporting Information (S1 Table through to and including S6 Table), which provide a more detailed description for the frequency of themes, the Member States that spoke to them, and income and regional groupings. For the quantitative observations in the thematic frequency analysis, all references to a specific theme/article were counted. For example, if the same Member State made two separate and substantively different references to an article, both were counted. For the frequency analysis concerning which Member States referenced an article, a Member State was only counted once even if they made multiple references to the same article in their intervention. Only the Member State delivering statements on behalf of a coalition were counted, rather than all the Member States that associated with the statement.

#### Qualitative analysis.

Qualitative analysis involved a deductive approach to coding of the transcribed data and using the article headings from Chapter II as a deductive code formulation for the identification of themes [34]. Key words and issues identified in manifest and literal content were used to support the coding process [34,38]. If LMIC statements referenced article number(s), and/or words or concepts in the article heading, and/or concepts or issues relevant to the substance of the article, it was coded and grouped thematically under relevant article heading(s). For example, if an intervention included ‘LMICs need increased access to research and development requiring predictable and sustainable financing’, the text was coded and organised under the relevant articles/themes, which are Article 9 (Research and development) and Article 20 (Sustainable financing). Data was extracted from the transcripts and organised in Microsoft Excel by theme/article, and by Member State. A range of published and publicly available sources were used to inform how external influences and events (exogenous factors), and internal attributes, circumstances and characteristics (endogenous factors) [39,40] contributed to LMIC expectations, positions and ambitions. As this research used published and/or publicly available data, ethics approval was not sought.

## Results

Initially presented is an overview of the quantitative observations for the articles LMICs accorded priority as informed by the frequency analysis. This is followed by a qualitative analysis that summarises the article and describes the issues and context central to LMIC positions and interests.

### Quantitative observations

Table 1 reflects a summary of the total frequency for references to each of the articles in Chapter II (A/INB/9/3) [32], as informed by S1 Table. Five articles attracted the greatest number of total references (n ≥ 35) informing the focus for the qualitative analysis. In descending order, this included: (i) Article 12: Access and benefit sharing (n = 58); (ii) Article 11: Transfer of technology and know-how (n = 53); (iii) Article 20: Sustainable financing (n = 53); (iv) Article 10: Sustainable and geographically diversified production (n = 42); (v) Article 9: Research and development (n = 35).

**Table 1:** Frequency of total references to articles by LMIC Member States during INB7, INB8, and INB9.

Article No.	Article Heading	Total
4	Pandemic prevention and surveillance	9
5	One Health approach to pandemic prevention preparedness and response	9
6	Preparedness, health system resilience and recovery	0
7	Health and care workforce	11
8	Preparedness, monitoring and functional reviews	0
9	Research and development	35
10	Sustainable and geographically diversified production	42
11	Transfer of technology and know-how	53
12	Access and benefit sharing	58
13	Supply chain and logistics	32
13 bis	National procurement–and distribution-related provisions	
14	Regulatory systems strengthening	2
15	Liability and compensation management	1
16	International collaboration and cooperation	6
17	Whole-of-government and whole-of-society approaches	1
18	Communication and public awareness	1
19	Implementation and support	24
20	Sustainable financing	53

Table 2 and Table 3, informed by S2 Table and S3 Table, respectively, illustrate the frequency of references to articles made by Member States disaggregated by income group, and WHO region, respectively. As this research reflects a point-in-time, and the webcasts are a partial insight into a much larger process, it is not possible to determine correlation. However, Table 3 suggests that upper-middle income countries were well represented during these INB sessions.

**Table 2:** References to articles by LMIC Member States disaggregated by World Bank income group.

INB	World Bank Income Group	Total
**INB7**	Low	11
	Lower-middle	26
	Upper-middle	56
**INB8**	Low	13
	Lower-middle	33
	Upper-middle	19
**INB9**	Low	24
	Lower-middle	42
	Upper-middle	56

The frequency of total references to articles by LMICs disaggregated by WHO region suggests that Member States from Africa were particularly active in the INB sessions within the research period.

**Table 3:** References to articles by LMIC Member States disaggregated by WHO Region.

INB	WHO Region	Total
**INB7**	Africa Region	36
	Region of the Americas	22
	Eastern Mediterranean Region	5
	European Region	4
	South-East Asia Region	17
	Western Pacific Region	9
**INB8**	Africa Region	32
	Region of the Americas	9
	Eastern Mediterranean Region	11
	European Region	0
	South-East Asia Region	7
	Western Pacific Region	3
**INB9**	Africa Region	42
	Region of the Americas	28
	Eastern Mediterranean Region	10
	European Region	0
	South-East Asia Region	21
	Western Pacific Region	16

### Qualitative findings

The articles that accorded the highest frequency of references are ones that LMIC Member States considered key to brokering the treaty and reflect contested issues in the multilateral system for global health. Four of the five articles are especially interrelated and interdependent as they focus on research and development (R&D), diversifying and increasing production capability, technology transfer and establishing a multilateral pathogen access and benefits system. The fifth article on sustainable financing is what LMICs consider an essential element to deliver on the treaty’s obligations. The illustrative examples below do not group or reference Member States beyond being LMICs. Also, as the order of the articles is important to their operationalisation, the analysis of articles/themes is presented sequentially.

#### Article 9: Research and development.

Article 9 is focused on increasing the R&D capacity and capability for public health and PPPR in LMICs. The article states that each Party shall develop policies for government-funded R&D agreements for PPPR that enable timely and equitable access via mechanisms including non-exclusive licensing, affordable pricing, and technology transfer on voluntary terms. A transparency measure compelling governments to publish the terms of government-funded R&D agreements during a pandemic is also included. LMICs were clear in articulating that the imbalance between the developed and developing states, as exemplified by access to COVID-19 vaccines, was a consequence of systemic constructs within the multilateral system. This article intends to contribute to increasing global production and supply of countermeasures by extending R&D and its benefits to more LMICs; however, this also presents enabling factors for some LMICs with manufacturing capability to support national objectives to grow their pharmaceutical sectors and markets.

*“…we want to underline that removing supply chain bottlenecks by facilitating transfer of technology and knowhow, access to R&D for geographical diversification for the production of vaccines and other pandemic related products, and access to therapeutics and diagnostics and health workforce, [and] removing non-tariff and other pandemic product barriers, as implied in certain articles, would be critical in successful implementation of the treaty.”* (India for South-East Asia Region, INB9)

LMICs are attuned to the complex landscape of R&D and understood the non-binding language on cooperation and knowledge-sharing, as well as the inability to compel key non-state actors in R&D (such as the private sector) fell short of their ambitions. Furthermore, as the article focuses on promoting government-funded investment, the issue of how much reform this article can catalyse remains pertinent given the treaty has negligible effect on the non-state R&D actors that LMICs consider important to their interests.

“*Governments investment in research and development to develop medical products including vaccines*, *diagnostics and medicines and finance thereof*, *from prevention and preparedness should be treated as global pandemic goods and accounted for as such.”* (Botswana, INB7)

#### Article 10: Sustainable and geographically diversified production.

Article 10 is concise in its intention to promote the sustainable production of pandemic products across LMICs in all regions. It is a core aspect of the treaty in view of ameliorating the supply shortfall of pandemic countermeasures, and LMICs are unequivocally emphatic that production capability must be extended to their territories and regions.

*“COVID*-*19 pandemic made it clear that diversified production of life*-*saving medicines is a need*, *not an aspiration. It showed the glaring gaps in existing financial mechanisms; it exposed the lack of solidarity within and among Member States at a time when we needed to be our sisters and brothers’ keepers; and exposed the lack of transparency and trust.”* (Eswatini, INB9)

LMICs also highlighted the importance of countermeasures being available to prevent a health emergency from turning into an outbreak, again reinforcing that the operationalisation of this article should ensure LMICs can source countermeasures quickly, and from national or regional suppliers.

*“…we did note the positive elements within this proposal* [*for*] *the inclusion of a separate article on sustainable production marks a significant advancement in our text. Enhancing local and regional production capacities is critical in preventing any disease outbreak and public health emergency from becoming a pandemic.”* (Pakistan, INB7)

#### Article 11: Transfer of technology and know-how.

Technology transfer (along with R&D) has a long history of contestation between developed and developing countries that underpins LMIC negotiating positions. While LMICs maintain that the substance of Article 11 is necessary for access to medicines and other essential health products, manufacturers and the developed states that host them often claim this would styme innovation. LMICs seize the opportunity to progress reform in the multilateral system, aligned with their expectations that the treaty support equity.

*“If we maintain the status quo in financing and access to technology and knowhow*, *transformation will never happen. It would be just and fair not to ask the developing countries to reinvent the wheel for saving lives of millions of people during public health emergencies and future pandemics.”* (Bangladesh, INB8)

However, the reference to existing frameworks such as the Doha Declaration on the Trade-Related Aspects of Intellectual Property Rights (TRIPS) Agreement and Public Health (2001) and use of non-compelling language signals this text does little to pressure developed states for concessions. In response, LMICs call-out the imbalanced application of compelling language, in view of the responsibility to equitably enable all Member States to deliver on treaty obligations and questioned the rhetorical commitment to PPPR when there was a lack of willingness to catalyse the reforms necessary to affect it.

*“Regarding the core equity articles in the text*, *articles 9*–*13*, *we welcome the retention of key text on intellectual property rights and transfer of technology and know*-*how. However*, *we note with concern the weak legal language and qualifiers used. In contrast*, *for example*, *to more legally binding language in Article 4 in pandemic prevention and public health surveillance*.” (Fiji, INB7)

#### Article 12: Access and benefit sharing.

This article focuses on the establishment of the multilateral WHO Pathogen Access and Benefit-Sharing System (PABS) that along with articles 9, 10 and 11, are central to the political-economy of the negotiations–given these articles are indicative of the scope of LMIC ambitions for the multilateral system–and the concessions required from developed states to realise them. The objective of PABS is to provide balanced, but transactional incentives, for sharing biological materials and genetic sequence data for early warning and the development of countermeasures. The article prescribes manufacturers will pay for access to data, provide a percentage of manufactured countermeasures at no-cost (10%) and not-for-profit (10%) to LMICs during a pandemic, and voluntarily contribute non-monetary benefits such as transfer of technology and capacity building.

*“We however note with great concern that Article 12 on pathogen access and benefits sharing that will translate the provisions on meaningful impact at all levels including in the community*, *lags behind*, *and there is no agreed legal text yet. We see this article and its linkages to the other articles as the backbone to the whole instrument…”* (Ethiopia, on behalf of the Africa Region and the Arab Republic of Egypt, INB 8)

The PABS proposal is an essential inclusion for LMICs in reaching agreement on the treaty. However, the non-compelling language reinforces the asymmetry LMICs want to realign through this process, particularly as they have been, and remain, an important source of biological and genetic material for outbreaks. Compounding this frustration is the omission of articulating PABS governance processes, raising concerns from LMICs that their expectations for influence in representational governance, critical to shaping the terms of the proposed PABS, will not be addressed.

“*We would also need to address the governance issue as highlighted by many countries. On pathogen access and benefit sharing*, *we believe that access and benefits need to be on equal footing. The obligation to share pathogens is not as critical as sharing the benefits arising from the use of those pathogens*, *especially in critical times..*.” (Indonesia, INB9)

#### Article 20: Sustainable financing.

LMIC Member States instigated the inclusion of this article that seeks to establish a coordinating financial mechanism to support implementation of the treaty and the International Health Regulations (2005). The article’s reference to a pooled fund within the United Nations system (to enable inclusive arrangements for Member States to participate in governance and funding decisions) is a priority for LMICs. This is in contrast to the mechanism also aiming to identify and coordinate existing financing for PPPR potentially including established funding sources outside the control of WHO and/or the pandemic treaty.

*“Sustainable financing is the key element to pandemic prevention and preparedness and response. Therefore*, *the Group emphasises the importance of establishing a sustainable financing mechanism that supports the implementation of the pandemic agreement. Taking into account the differences in capacities and capabilities among member states.“* (Bangladesh, for the Group for Equity, INB9)

The inclusion of the pooled fund indicates the high-level of coordination across LMICs as supported by regional and principles-based coalitions, such as the Group for Equity, which has bolstered the influence of LMICs in these negotiations. The quantum of resources is important, however, the arrangements for how and who controls the fund, including allocation decisions, is fundamental. LMICs have a much better chance of inclusive governance if financing resources are included under the auspices of the treaty, WHO and/or United Nations system (compared to coopting mechanisms outside of the treaty’s influence).

The different capacities and levels of socio-economic development across LMICs suggest some will be better placed to access the potential benefits stemming from the treaty. However, despite the prospect of an uneven flow of benefits, LMICs maintain the rhetoric that equity must underpin the treaty process, its operationalisation and outcomes. The burden of treaty obligations coupled with the perceived unwillingness from developed states to make concessions is central to LMIC concerns, and in practical terms this involves rebalancing the distribution, and access to, financial resources for PPPR.

*“….many of the obligations we are going to create through this instrument … are already met mostly by developed countries… you do not have to do all the work as us developing countries … the finish line is further ahead for us in terms of what we have to build.”* (Brazil, INB7)

## Discussion

An important preface to the discussion of key articles and the factors that contribute to LMIC positions is situating the work of The Panel, which prioritises equity and many of the principles of a cosmopolitan and globalist approach that helps in contextualising the coordinated positions of LMICs. This background provides context for the following discussion of LMIC positions, and the exogenous and endogenous factors that help to explain these.

The initial report and recommendations of The Panel [3] is an important lens through which to consider LMIC positions, and underscores how some of the endogenous and exogenous factors that informed these positions are interrelated. The Panel’s analysis and recommendations function as a measure of accountability highlighting inequity as both cause and consequence of what resulted during COVID-19. What it brings to bare is that states acting in their own interests in line with a realist approach to health security [7,9,41] does not enable effective PPPR if the operating premise is "no one is safe until everyone is safe" [42]. While the Panel’s lessons, recommendations and considerations of equity focus on the COVID-19 pandemic, many of the ideas, issues and principles have been reflected in global health, global health security and PPPR commentary and analysis prior to the COVID-19 pandemic. The negotiations exemplify that reducing inequity will challenge structural economic and political constructs [43] given the ask of for Member States who have “more capacities and resources relevant to pandemics” [12] to contribute more than those that have less. As acknowledged by The Panel and others, the zero-sum game [44,45] of ‘have’ and ‘have-nots’ for vaccine access was considered a moral and ethical failure that undermined global PPPR efforts. The Panel also asserted that a stronger and equitable multilateral PPPR system can only be achieved if it is premised on a cosmopolitan [4–6] and globalist approach [7,9,46,47]. WHO similarly purports this, given it depends on voluntary actions and soft power to achieve the multilateral cooperation the treaty process and outcome depends on [7,8,46,48]. As such, LMICs have leaned-into the principles of global solidarity and cosmopolitanism, and the existence of these frameworks present key exogenous factors helping to explain their positions.

Some LMICs have used the treaty negotiations to simultaneously prosecute national ambitions whilst asserting collective positions that promote global solidarity and equity. While the potential for all LMICs to benefit equitably through treaty provisions is unlikely, common to LMIC interests is leveraging change in the multilateral system to rebalance power and capacity away from developed states. This has resonance with Sabatier’s Advocacy Coalition Framework [49–51] that acknowledges the effect of broader external factors in influencing coalition behaviour and collective positions. The backdrop to the treaty negotiations of geopolitical competition, polycrisis and conflict [52] has reinforced the asymmetry of political and economic power between developed and developing states. During the research period this included the conflict in Gaza, which featured in LMIC statements. This too contributes to the collective positions taken by LMICs, as strengthened by regional and principles-based coalitions [20] such as the 29 Member States [53] comprising the Group for Equity [54,55], which has helped LMICs gain traction on the contested articles discussed below. As the negotiations progress, more granular detail on LMIC positions, and the positions of developed states in comparison, could be useful to explore in future research.

### R&D, diversified production, and transfer of technology and know-how

Common and core to enabling Articles 9, 10 and 11 is intellectual property (IP) rights for medicines, vaccines, diagnostics and other health products, to which the 2001 Doha Declaration on TRIPS and Public Health [56] is central. TRIPS flexibilities are to preserve and promote public health; for example, by permitting Governments to access patented products without the IP holder’s consent [57]. India and South Africa are prominent voices in the negotiations, for their regions and as members of the Group for Equity. In 2020, India and South Africa jointly sought a TRIPS waiver to expand access to vaccines, therapeutics and diagnostics.

India and South Africa’s TRIPS waiver request of 2020 [58] to the World Trade Organization (WTO) is important to understanding their respective positions, and the exogenous and endogenous factors that help to explain them. The initial request sought to enable the production and supply of COVID-19 vaccines at a time of dire supply shortfall. In May 2021, India and South Africa updated the waiver request to include therapeutics and diagnostics (with the co-sponsorship of 65 LMIC Member States [59,60]. In June 2022, when a decision was made to override patent rights to produce COVID-19 vaccines for export, it was described by several LMICs as a “narrow-conditioned Decision due to demands of some WTO Members” [61], thus criticising the developed states that had initially objected to it. The waiver request for therapeutics and diagnostics was only concluded in February 2024, when the TRIPS Council advised no consensus could be reached [57].

The outcome of the TRIPS waiver requests [57] were perceived by LMICs as wealthy states again influencing the multilateral system to monopolise production and supply of countermeasures, despite a pandemic [62]. However, also relevant is India’s decision in 2021 to curb its exports of COVID-19 vaccines due to the Delta variant outbreak, whereby it similarly exercised national interests like other vaccine producers including the European Union [63]. Nevertheless, the recency of India and South Africa’s waiver request relative to the treaty negotiations influences and legitimises their positions on Articles 9, 10 and 11 from the perspective of driving global equity, as well as enabling them to re-prosecute issues important to their national interests for pharmaceutical manufacturing.

Endogenous factors that explain India and South Africa’s positions concern their significant pharmaceutical production capability. India is the world’s third largest producer and a leading exporter of generic medicines and vaccines with an annual production value of around $US 43 billion (2022–2023) [64]. South Africa has developed its local manufacturing industry, which is the largest manufacturer and supplier of pharmaceuticals in sub-Saharan Africa [65]. It is also home to WHO’s Global mRNA Vaccine Technology Transfer Hub [66] and has ambitions to establish a state-owned pharmaceutical company to support its national health insurance program [67].

The negotiation of these articles presents an opportunity to progress national interests both Member States have previously tried to advance in the WTO. While the treaty is under the auspices of WHO, The Panel notes the potential “important political and strategic value to encourage pharmaceutical firms” [68] to engage in voluntary licensing and transfer of technology and know-how. However, India and South Africa have little need to make this explicit; the rhetorical power of their argument lies in premising equity and a cosmopolitan approach for the concessions and reforms these articles could trigger for LMICs, even if the benefits do not flow through to all LMICs equally.

### Access and benefit sharing

The biological data sharing under the PABS proposal has several precedent instruments which have called for and/or informed pathogen sharing arrangements. These have initially emphasized the environment and biotrade, and include the Convention on Biological Diversity (1992), International Health Regulations (2005), Nagoya Protocol on Access to Genetic Resources and the Fair and Equitable Sharing of Benefits Arising from their Utilization (2010) and The Pandemic Influenza Preparedness Framework (2011) [69] (influenced by Indonesia’s avian influenza outbreak in 2005 [70]). The various experiences of LMICs in PABS suggests the enormous complexity underlying how countries contributing to pathogen and genetic material sharing equitably share in the benefits [12]. Furthermore, the multilateral PABS proposed under the pandemic treaty will exist amidst already established national policies, guidelines and legislations that has given rise to a myriad of arrangements [71].

For example, Brazil and Namibia are both strong advocates on PABS and have national legislation in place for access and benefit sharing [71–73]. While their respective systems recognise monetary and non-monetary benefits, legislation alone does not ensure equitable benefit sharing. Brazil’s economic, technological and knowledge capability, particularly through public investment in health and pharmaceutical production [74] supports a stronger position in negotiating benefit sharing given Brazil’s objectives to utilise and bolster existing pharmaceutical, R&D and countermeasures capabilities [71]. In contrast, communities in Namibia from which samples were extracted have not always equitably benefitted [73] due to the asymmetry of benefit sharing knowledge and capabilities favouring those with political and economic advantage.

Like Brazil and Namibia, Indonesia is active in these negotiations including on behalf of the Group for Equity. Indonesia has explicitly stated that the details of governance arrangements and benefit sharing must also be addressed [75] in the treaty for there to be agreement. Their position stresses the need to compel the provision of benefits, allocate countermeasures based on need rather than purchasing power, and to include LMICs in PABS governance and decision-making processes. The experience of Indonesia during an avian influenza outbreak helps explain the exogenous and endogenous factors contributing to their negotiating position.

Indonesia’s experience during the H5N1 avian influenza outbreaks (2005–2007) culminated in the Government of Indonesia discontinuing to sample-share with the WHO system, for reasons of inequitable access to benefits [70,76,77]. The virus samples Indonesia had shared with WHO reference laboratories were subject to breaches of WHO guidelines, including the presentation of data internationally, and access to it by manufacturers for vaccine production, both occurring without Indonesia’s prior knowledge and consent [76]. Despite being the worst affected country during this outbreak [76], Indonesia was unsuccessful in securing timely access to vaccines from WHO, or commercially, due to the advance purchase commitments made by the manufacturers [70]. Indonesia defended its case at the World Health Assembly in 2007 [78] and subsequently introduced the concept of ‘viral sovereignty’ over its pathogen samples [77] and challenged global health governance regarding the inequities in access to benefits for data shared by developing countries [76,79,80].

Endogenous factors contributing to Indonesia’s position is their understanding of the power and sensitivity of sample data to enable early warning, development of countermeasures [77], and the potential to catalyse negative impacts such as border closures [80,81]. Moreover, Indonesia has the largest pharmaceutical sector in Southeast Asia, which services a growing domestic market and national health insurance program [82]. Its state-owned and commercial pharmaceutical producers are important to its economic and health ambitions [82]. Exogenous factors informing Indonesia’s position concern reform of the multilateral system to even-up the benefits, access and influence for LMICs. However, underlying Indonesia’s rhetoric is their balancing (but not abandoning) of national interests and sovereignty to leverage the negotiations for state-centric objectives, including access and benefit sharing.

### Sustainable financing

LMICs have a shared position on the inclusion of sustainable financing in the text being critical toward progressing agreement. However, these negotiations have raised key questions about the shape of sustainable financing under this treaty. For example, what will it support (PPPR and surge financing), what modalities will be included (such as debt swap arrangements and technical support), what triggers financing support, and can newly established funds such as The Pandemic Fund be brought under the treaty [12,83]. The Panel determined there were insufficient funding levels for preparedness, and funds were too slow to come on-stream when the pandemic was declared [3]. The Panel consequently recommended the creation of a pandemic preparedness financing facility to mobilise $US 5–10 billion per annum (whereby developed countries pay more) and having $US 50–100 billion to rapidly disburse on declaration of a pandemic [3].

Exogenous factors contributing to LMIC positions concern the interdependency of global PPPR on the capacity and capability of national systems [1] and the competition for PPPR resources during a pandemic, which requires rapid access to financing. LMIC positions iterate that PPPR financing needs to be predictable and accessible during pandemic and inter-pandemic periods. This is to remedy what occurred during COVID-19, whereby WHO and the UN Central Emergency Response Fund were significantly unprepared, having only allocated $US 24 million one-month after the pandemic was declared. Similarly, the World Bank’s insurance and capital-market instruments took three-months to come on-stream with initially $US 196 million to be shared across 64 countries [84].

The response to inject financing for PPPR was met with The Pandemic Fund established in late 2022 [85] under the auspices of the World Bank with initially fifteen member state entities and three philanthropies as donors [86], which also falls far short of the quantum, flexibility and equity The Panel recommended, and that LMICs are seeking [87]. The proposal that The Pandemic Fund–not considered by LMICs to have inclusive representation and governance [12,88] –raises questions about how an established fund external to WHO and/or the treaty process could be integrated into a WHO led PPPR response under the auspices of the treaty. Furthermore, a single fund rather than a systemic approach to harmonise and streamline access to financing and financial support [89] resonates with early critique that an additional fund contributes to an already fractured global health financing system [83,87].

Endogenous factors informing LMIC positions involve country and health system context and respective experiences of COVID-19. Ethiopia, a low-income country that has spoken for the Africa Region and the Group of Equity, is a key interlocutor. With a population of around 126 million people and extremely uneven levels of development across the country [90], health sector challenges include financial and health workforce shortages, poor infrastructure and limited means to buffer social and economic shocks, such as outbreaks, drought, extreme weather and conflict [91]. It has one of the highest rates of child malnutrition globally; only 60 per cent of the population access safe drinking water and less than 30 per cent have access to sanitation services (2021) [90–93]. COVID-19, drought, disease outbreaks, conflict, debt and inflation have setback health and nutrition services and exacerbated food insecurity and poverty [90,92]. Given the resourcing gaps and strain on Ethiopia’s health system, negotiating for additional, timely and flexible funding to deliver treaty obligations is a rational position.

### Strengths and Limitations

The strengths of this research include looking in detail at the key ‘equity asks’ voiced by individual LMICs during pandemic treaty negotiations. It also illustrates that the strategies of some LMICs to pursue multiple objectives accommodated under the headline of global solidarity, providing insight into their positions and highlights key factors underpinning collective action and coalition behaviour in negotiations. This work also contributes to understanding how PPPR, global health diplomacy and multilateral cooperation continue to be influenced and shaped by the treaty process going forward.

The limitations involve this research being at a point-in-time and not capturing the breadth of the negotiation process. Rather, it offers insight during a key negotiating period of the continuing pandemic treaty process. It is also acknowledged Member State statements and interventions do not reveal the breadth of influencing factors nor full rationale for their positioning in these webcast negotiations. In this view, only observations are made and no correlation is asserted. Further, the data used for this research was limited to sources in English. This includes drawing on WHO’s interpretation services for the webcast INB negotiation sessions. As data saturation was not an objective, the quotes included are intended to be illustrative rather than exhaustive. As noted earlier, more granular description of LMIC positions including expansion of the data set to key informant interviews and/or desk review could be useful to explore in future research. Most importantly, it is acknowledged that this work was prepared by a researcher working in, and originating from, a developed country. There is explicit recognition this work does not fully capture the perceptions or opinions of the LMICs involved in the negotiations.

## Conclusion

This window into negotiations at a critical juncture of the pandemic treaty process reveals that LMICs present collective positions for which equity is the headline, and a means, to prosecute multiple objectives on the global stage. The coordination of LMICs during negotiations has enabled traction on the discussion of reforms to the multilateral health system, including where there has been limited prior success. LMIC positions reflect and reinforce The Panel’s assessments and recommendations that support a narrative of equity being synonymous with effective PPPR [94]. Additionally, these negotiations have presented opportunities for some LMICs to pursue national ambitions without needing to break, or being isolated from, collective positions and coalitions. This does not infer that LMICs are not cognisant of the national interests some seek to prosecute. Rather, it is an indication of the priority placed on the common ambition to ensure the treaty does not drive further inequity between developing and developed Member States in the global health system.

As the discussion of PPPR in multilateral global health fora continues, how the treaty is progressed sends an important signal about the value Member States place on the multilateral system to address collective challenges and the confidence placed in WHO to coordinate, safeguard and deliver PPPR. This research suggests that LMIC agreement to the proposed treaty hinges on concessions from developed states. Further, the nature and scope of concessions from developed states will also be influenced by the degree to which LMICs continue to maintain collective positions to nudge the multilateral system in the direction of equity, and the response from developed states during remaining treaty negotiations.

## Supporting information

S1 Table Frequency of references to each of the Articles by LMIC Member States during INB7, INB8, and INB9 webcast sessions. (DOCX)

S2 Table Frequency of references to each of the Articles by LMIC Member States during INB7, INB8, and INB9 webcast sessions, disaggregated by income group. (DOCX)

S3 Table Frequency of references to each of the Articles by LMIC Member States during INB7, INB8, and INB9 webcast sessions, disaggregated by WHO Region. (DOCX)

S4 Table References to Articles by LMIC Member States (including income and regional group) during INB7 webcast sessions. (DOCX)

S5 Table References to Articles by LMIC Member States (including income and regional group) during INB8 webcast sessions. (DOCX)

S6 Table References to Articles by LMIC Member States (including income and regional group) during INB9 (18–28 March) webcast sessions. (DOCX)
