# Supplementary material for: Understanding the expectations, positions and ambitions of LMICs during pandemic treaty negotiations, and the factors contributing to them
Source: PLOS Glob Public Health. 2025 Mar 12;5(3):e0003851. doi: 10.1371/journal.pgph.0003851 (PMC11902204; doi:10.1371/journal.pgph.0003851)
Supplement: S1 Table — (DOCX) [file pgph.0003851.s001.docx]

**S1 Table. Frequency of references to each of the Articles by LMIC Member States during INB7, INB8, and INB9 webcast sessions.**

|  | **Article  4** | **Article  5** | **Article  6** | **Article  7** | **Article  8** | **Article  9** | **Article  10** | **Article  11** | **Article  12** | **Article  13**  **13 bis** | **Article  14** | **Article  15** | **Article  16** | **Article  17** | **Article  18** | **Article  19** | **Article  20** |
| --- | --- | --- | --- | --- | --- | --- | --- | --- | --- | --- | --- | --- | --- | --- | --- | --- | --- |
|  | **Pandemic prevention and surveillance** | **One Health approach to pandemic prevention, preparedness and response** | **Preparedness, health system resilience and recovery** | **Health and care workforce** | **Preparedness, monitoring and functional reviews** | **Research and development** | **Sustainable and geographically diversified production** | **Transfer of technology and know-how** | **Access and benefit sharing** | **Supply chain and logistics National procurement - and distribution-related provisions** | **Regulatory systems strengthening** | **Liability and compensation management** | **International collaboration and cooperation** | **Whole-of-government and whole-of-society approaches** | **Communication and public awareness** | **Implementation and support** | **Sustainable financing** |
| **INB7** | 2 | 4 | 0 | 4 | 0 | 18 | 12 | 18 | 20 | 14 | 0 | 0 | 2 | 1 | 0 | 5 | 14 |
| **INB8** | 2 | 0 | 0 | 3 | 0 | 5 | 13 | 17 | 15 | 6 | 0 | 0 | 2 | 0 | 0 | 6 | 16 |
| **INB9** | 5 | 5 | 0 | 4 | 0 | 12 | 17 | 18 | 23 | 12 | 2 | 1 | 2 | 0 | 1 | 13 | 23 |
| **Total** | **9** | **9** | **0** | **11** | **0** | **35** | **42** | **53** | **58** | **32** | **2** | **1** | **6** | **1** | **1** | **24** | **53** |

**Notes:**

- Article headings are based on *A/INB/9/3: Revised draft of the negotiating text of the WHO Pandemic Agreement, 13 March 2024*, <https://apps.who.int/gb/inb/pdf_files/inb9/A_inb9_3-en.pdf>.
