# Supplementary material for: Understanding the expectations, positions and ambitions of LMICs during pandemic treaty negotiations, and the factors contributing to them
Source: PLOS Glob Public Health. 2025 Mar 12;5(3):e0003851. doi: 10.1371/journal.pgph.0003851 (PMC11902204; doi:10.1371/journal.pgph.0003851)
Supplement: S2 Table — (DOCX) [file pgph.0003851.s002.docx]

**S2 Table. Frequency of references to each of the Articles by LMIC Member States during INB7, INB8, and INB9 webcast sessions, disaggregated by income group.**

|  | |  | **Article  4** | **Article  5** | **Article  6** | **Article  7** | **Article  8** | **Article  9** | **Article  10** | **Article  11** | **Article  12** | **Article  13 & 13 bis** | **Article  14** | **Article  15** | **Article  16** | **Article  17** | **Article  18** | **Article  19** | **Article  20** | **Total** |
| --- | --- | --- | --- | --- | --- | --- | --- | --- | --- | --- | --- | --- | --- | --- | --- | --- | --- | --- | --- | --- |
|  |  |  | **Pandemic prevention and surveillance** | **One Health approach to pandemic prevention, preparedness and response** | **Preparedness, health system resilience and recovery** | **Health and care workforce** | **Preparedness, monitoring and functional reviews** | **Research and development** | **Sustainable and geographically diversified production** | **Transfer of technology and know-how** | **Access and benefit sharing** | **Supply chain and logistics National procurement - & distribution-related provisions** | **Regulatory systems strengthening** | **Liability and compensation management** | **International collaboration and cooperation** | **Whole-of-government and whole-of-society approaches** | **Communication and public awareness** | **Implementation and support** | **Sustainable financing** |  |
| **INB7** | **Low** | | 0 | 1 | 0 | 1 | 0 | 1 | 2 | 2 | 2 | 1 | 0 | 0 | 0 | 0 | 0 | 0 | 1 | **11** |
|  | **Lower-middle** | | 1 | 1 | 0 | 1 | 0 | 4 | 2 | 4 | 4 | 3 | 0 | 0 | 1 | 0 | 0 | 2 | 3 | **26** |
|  | **Upper-middle** | | 1 | 2 | 0 | 2 | 0 | 8 | 7 | 10 | 8 | 7 | 0 | 0 | 1 | 1 | 0 | 3 | 6 | **56** |
| **INB8** | **Low** | | 0 | 0 | 0 | 0 | 0 | 1 | 2 | 1 | 2 | 2 | 0 | 0 | 0 | 0 | 0 | 2 | 3 | **13** |
|  | **Lower-middle** | | 0 | 0 | 0 | 0 | 0 | 2 | 4 | 8 | 6 | 2 | 0 | 0 | 2 | 0 | 0 | 2 | 7 | **33** |
|  | **Upper-middle** | | 0 | 0 | 0 | 0 | 0 | 1 | 4 | 2 | 5 | 3 | 0 | 0 | 0 | 0 | 0 | 2 | 2 | **19** |
| **INB9** | **Low** | | 0 | 0 | 1 | 1 | 0 | 2 | 3 | 3 | 3 | 3 | 1 | 0 | 0 | 0 | 0 | 3 | 4 | **24** |
|  | **Lower-middle** | | 3 | 2 | 2 | 2 | 0 | 3 | 5 | 5 | 6 | 2 | 0 | 1 | 1 | 0 | 1 | 3 | 6 | **42** |
|  | **Upper-middle** | | 2 | 2 | 2 | 1 | 0 | 4 | 7 | 8 | 9 | 6 | 1 | 0 | 1 | 0 | 0 | 5 | 8 | **56** |

**Notes:**

- Income groups is per World Bank Country and Lending Groups (2024 fiscal year) (<https://datahelpdesk.worldbank.org/knowledgebase/articles/906519-world-bank-country-and-lending-groups>)
