# Supplementary material for: Understanding the expectations, positions and ambitions of LMICs during pandemic treaty negotiations, and the factors contributing to them
Source: PLOS Glob Public Health. 2025 Mar 12;5(3):e0003851. doi: 10.1371/journal.pgph.0003851 (PMC11902204; doi:10.1371/journal.pgph.0003851)
Supplement: S3 Table — (DOCX) [file pgph.0003851.s003.docx]

**S3 Table. Frequency of references to each of the Articles by LMIC Member States during INB7, INB8, and INB9 webcast sessions, disaggregated by WHO Region.**

|  |  | **Article 4** | **Article 5** | **Article 6** | **Article 7** | **Article 8** | **Article 9** | **Article 10** | **Article 11** | **Article 12** | **Article 13  13 bis** | **Article 14** | **Article 15** | **Article 16** | **Article 17** | **Article 18** | **Article 19** | **Article 20** | **Total** |
| --- | --- | --- | --- | --- | --- | --- | --- | --- | --- | --- | --- | --- | --- | --- | --- | --- | --- | --- | --- |
|  |  | **Pandemic prevention and surveillance** | **One Health approach to pandemic prevention, preparedness and response** | **Preparedness, health system resilience and recovery** | **Health and care workforce** | **Preparedness, monitoring and functional reviews** | **Research and development** | **Sustainable and geographically diversified production** | **Transfer of technology and know-how** | **Access and benefit sharing** | **Supply chain and logistics National procurement - and distribution-related provisions** | **Regulatory systems strengthening** | **Liability and compensation management** | **International collaboration and cooperation** | **Whole-of-government and whole-of-society approaches** | **Communication and public awareness** | **Implementation and support** | **Sustainable financing** |  |
| **INB7** | Africa | 0 | 1 | 0 | 2 | 0 | 6 | 4 | 6 | 6 | 5 | 0 | 0 | 0 | 0 | 0 | 2 | 4 | **36** |
|  | Americas | 1 | 1 | 0 | 0 | 0 | 3 | 3 | 5 | 3 | 2 | 0 | 0 | 0 | 0 | 0 | 1 | 3 | **22** |
|  | Eastern Mediterranean | 0 | 0 | 0 | 0 | 0 | 0 | 1 | 1 | 1 | 0 | 0 | 0 | 0 | 0 | 0 | 0 | 2 | **5** |
|  | European | 0 | 0 | 0 | 0 | 0 | 1 | 1 | 1 | 1 | 0 | 0 | 0 | 0 | 0 | 0 | 0 | 0 | **4** |
|  | South-East Asia | 1 | 1 | 0 | 1 | 0 | 2 | 1 | 2 | 2 | 2 | 0 | 0 | 2 | 0 | 0 | 2 | 1 | **17** |
|  | Western Pacific | 0 | 1 | 0 | 1 | 0 | 1 | 1 | 1 | 1 | 2 | 0 | 0 | 0 | 1 | 0 | 0 | 0 | **9** |
| **INB8** | Africa | 0 | 0 | 0 | 0 | 0 | 3 | 5 | 5 | 5 | 3 | 0 | 0 | 1 | 0 | 0 | 4 | 6 | **32** |
|  | Americas | 0 | 0 | 0 | 0 | 0 | 0 | 3 | 0 | 3 | 2 | 0 | 0 | 0 | 0 | 0 | 1 | 0 | **9** |
|  | Eastern Mediterranean | 0 | 0 | 0 | 0 | 0 | 1 | 1 | 2 | 2 | 0 | 0 | 0 | 1 | 0 | 0 | 1 | 3 | **11** |
|  | European | 0 | 0 | 0 | 0 | 0 | 0 | 0 | 0 | 0 | 0 | 0 | 0 | 0 | 0 | 0 | 0 | 0 | **0** |
|  | South-East Asia | 0 | 0 | 0 | 0 | 0 | 0 | 1 | 2 | 2 | 0 | 0 | 0 | 0 | 0 | 0 | 0 | 2 | **7** |
|  | Western Pacific | 0 | 0 | 0 | 0 | 0 | 0 | 0 | 1 | 1 | 1 | 0 | 0 | 0 | 0 | 0 | 0 | 0 | **3** |
| **INB9** | Africa | 0 | 0 | 2 | 1 | 0 | 4 | 5 | 5 | 6 | 5 | 1 | 0 | 1 | 0 | 0 | 4 | 8 | **42** |
|  | Americas | 2 | 2 | 2 | 0 | 0 | 1 | 5 | 5 | 5 | 3 | 0 | 0 | 0 | 0 | 0 | 1 | 2 | **28** |
|  | Eastern Mediterranean | 0 | 0 | 0 | 0 | 0 | 0 | 1 | 2 | 1 | 1 | 1 | 0 | 0 | 0 | 0 | 2 | 2 | **10** |
|  | European | 0 | 0 | 0 | 0 | 0 | 0 | 0 | 0 | 0 | 0 | 0 | 0 | 0 | 0 | 0 | 0 | 0 | **0** |
|  | South-East Asia | 1 | 1 | 0 | 2 | 0 | 2 | 3 | 3 | 3 | 1 | 0 | 0 | 0 | 0 | 0 | 2 | 3 | **21** |
|  | Western Pacific | 1 | 1 | 1 | 1 | 0 | 1 | 1 | 1 | 3 | 1 | 0 | 1 | 1 | 0 | 1 | 0 | 2 | **16** |

**Notes:**

- WHO Regions: Africa Region; Region of the Americas; Eastern Mediterranean Region; European Region; South-East Asia Region; and Western Pacific Region (<https://www.who.int/countries/>).
