# Supplementary material for: Understanding the expectations, positions and ambitions of LMICs during pandemic treaty negotiations, and the factors contributing to them
Source: PLOS Glob Public Health. 2025 Mar 12;5(3):e0003851. doi: 10.1371/journal.pgph.0003851 (PMC11902204; doi:10.1371/journal.pgph.0003851)
Supplement: S5 Table — (DOCX) [file pgph.0003851.s005.docx]

**S5 Table. References to Articles by LMIC Member States (including income and regional group) during INB8 webcast sessions.**

| **Article 4** | **Article 5** | **Article 6** | **Article 7** | **Article 8** | **Article 9** | **Article 10** | **Article 11** | **Article 12** | **Article 13** | **Article 14** | **Article 15** | **Article 16** | **Article 17** | **Article 18** | **Article 19** | **Article 20** |
| --- | --- | --- | --- | --- | --- | --- | --- | --- | --- | --- | --- | --- | --- | --- | --- | --- |
| **Pandemic prevention and public health surveillance** | **One Health** | **Preparedness, readiness and resilience** | **Health and care workforce** | **Preparedness, monitoring and functional reviews** | **Research and development** | **Sustainable production** | **Transfer of technology and know-how** | **Access and benefit sharing** | **Global Supply Chain and Logistics Network** | **Regulatory strengthening** | **Compensation and liability management** | **International collaboration and cooperation** | **Whole-of-government and whole-of-society approaches at the national level** | **Communication and public awareness** | **Implementation capacities and support** | **Financing** |
| **0** | **0** | **0** | **0** | **0** | **4** | **10** | **11** | **13** | **6** | **0** | **0** | **2** | **0** | **0** | **6** | **12** |
|  |  |  |  |  | **Egypt**  (LM) (EM) | **Mexico**  (UM) (AM) | **Egypt**  (LM) (EM) | **Mexico**  (UM) (AM) | **Mexico**  (UM) (AM) |  |  | **Bangladesh**  (LM) (SEA) |  |  | **Mozambique**  (L) (AF) | **Egypt**  (LM) (EM) |
|  |  |  |  |  | **South Africa**  (UM) (AF) | **Egypt**  (LM) (EM) | **Ethiopia**  (L) (AF) | **Egypt**  (LM) (EM) | **Mozambique**  (L) (AF) |  |  | **Indonesia**  (UM) (SEA) |  |  | **Ecuador**  (UM) (AM) | **Ethiopia**  (L) (AF) |
|  |  |  |  |  | **Nigeria**  (LM) (AF) | **Ethiopia**  (L) (AF) | **Zambia**  (LM) (AF) | **Ethiopia**  (L) (AF) | **South Africa**  (UP) (AF) |  |  |  |  |  | **South Africa** (UM) (AF) | **Bangladesh**  (LM) (SEA) |
|  |  |  |  |  | **Ethiopia**  (L) (AF) | **Bangladesh**  (LM) (SEA) | **Eswatini**  (LM) (AF) | **Bangladesh**  (LM) (SEA) | **Brazil**  (UM) (AM) |  |  |  |  |  | **Nigeria**  (LM) (AF) | **Zambia**  (LM) (AF) |
|  |  |  |  |  |  | **Eswatini**  (LM) (AF) | **South Africa**  (UM) (AF) | **China**  (UM) (WP) | **Philippines**  (LM) (WP) |  |  |  |  |  | **Pakistan**  (LM) (EM) | **Eswatini**  (LM) (AF) |
|  |  |  |  |  |  | **Mozambique**  (L) (AF) | **Philippines**  (LM) (WP) | **Eswatini**  (LM) (AF) | **Ethiopia**  (L) (AF) |  |  |  |  |  | **The Gambia**  (L) (AF) | **Mozambique**  (L) (AF) |
|  |  |  |  |  |  | **Ecuador**  (UM) (AM) | **Palestine**  (UM) (EM) | **Mozambique**  (L) (AF) |  |  |  |  |  |  |  | **South Africa**  (UM) (AF) |
|  |  |  |  |  |  | **South Africa** (UM) (AF) | **Nigeria**  (LM) (AF) | **Ecuador**  (UM) (AM) |  |  |  |  |  |  |  | **Palestine**  (UM) (EM) |
|  |  |  |  |  |  | **Brazil**  (UM) (AM) | **Pakistan**  (LM) (SEA) | **South Africa** (UM) (AF) |  |  |  |  |  |  |  | **Nigeria**  (LM) (AF) |
|  |  |  |  |  |  | **Nigeria**  (LM) (AF) | **India**  (LM) (SEA) | **Brazil**  (UM) (AM) |  |  |  |  |  |  |  | **Pakistan**  (LM) (EM) |
|  |  |  |  |  |  |  | **Bangladesh**  (LM) (SEA) | **Nigeria**  (LM) (AF) |  |  |  |  |  |  |  | **India**  (LM) (SEA) |
|  |  |  |  |  |  |  |  | **Pakistan**  (LM) (EM) |  |  |  |  |  |  |  | **The Gambia**  (L) (AF) |
|  |  |  |  |  |  |  |  | **India**  (LM) (SEA) |  |  |  |  |  |  |  |  |
|  |  |  |  |  |  |  |  |  |  |  |  |  |  |  |  |  |
|  |  |  |  |  |  |  |  |  |  |  |  |  |  |  |  |  |
|  |  |  |  |  |  |  |  |  |  |  |  |  |  |  |  |  |

**Notes:**

- Article headings are based on *A/INB/7/3, Proposal for negotiating text of the WHO Pandemic Agreement, 30 October 2023* (<https://apps.who.int/gb/inb/pdf_files/inb7/A_INB7_3-en.pdf>).
- Income groups is per World Bank Country and Lending Groups (2024 fiscal year) (<https://datahelpdesk.worldbank.org/knowledgebase/articles/906519-world-bank-country-and-lending-groups>)
  - **Upper-middle income = (UM); Lower-middle income = (LM); Low income = (L)**
- WHO Regions: Africa Region **(AF)**; Region of the Americas **(AM)**; Eastern Mediterranean Region **(EM)**; European Region **(EUR)**; South-East Asia Region **(SEA)**; and Western Pacific Region **(WP)** (<https://www.who.int/countries/>).
- A Member State is only counted once under each Article, even if they made more than one reference to the Article during their intervention.
- Mexico delivered a statement on behalf of Americas Region.
- Egypt delivered a statement on behalf of the Group for Equity; Pakistan delivered a statement on behalf of the Group for Equity (Closing session).
- Ethiopia delivered a statement on behalf of the 47 Member States of the African Region and the Arab Republic of Egypt.
- India delivered a statement on behalf of South-East Asia.
