# Supplementary material for: Understanding the expectations, positions and ambitions of LMICs during pandemic treaty negotiations, and the factors contributing to them
Source: PLOS Glob Public Health. 2025 Mar 12;5(3):e0003851. doi: 10.1371/journal.pgph.0003851 (PMC11902204; doi:10.1371/journal.pgph.0003851)
Supplement: S6 Table — (DOCX) [file pgph.0003851.s006.docx]

**S6 Table.** **References to Articles by LMIC Member States (including income and regional group) during INB9 (18-28 March) webcast sessions.**

| **Article 4** | **Article 5** | **Article 6** | **Article 7** | **Article 8** | **Article 9** | **Article 10** | **Article 11** | **Article 12** | **Article 13 & *13 bis*** | **Article 14** | **Article 15** | **Article 16** | **Article 17** | **Article 18** | **Article 19** | **Article 20** |
| --- | --- | --- | --- | --- | --- | --- | --- | --- | --- | --- | --- | --- | --- | --- | --- | --- |
| **Pandemic prevention and** *~~public~~ ~~health~~* **surveillance** | **One Health** *approach to pandemic prevention, preparedness and response* | **Preparedness***, health system* *resilience and recovery* | **Health and care workforce** | **Preparedness, monitoring and functional reviews** | **Research and development** | **Sustainable** *and geographically diversified production* | **Transfer of technology and know-how** | **Access and benefit sharing** | **Supply chain & logistics** *National procurement – and distribution-related provisions* | **Regulatory** *systems* **strengthening** | *Liability and* **compensation management** | **International collaboration and cooperation** | **Whole-of-government and whole-of-society approaches ~~at the national level~~** | **Communication and public awareness** | **Implementation capacities and support** | *Sustainable* **financing** |
| **5** | **4** | **5** | **4** | **0** | **9** | **15** | **16** | **18** | **11** | **2** | **1** | **2** | **0** | **1** | **11** | **18** |
| **Brazil**  (UM) (AM) | **Brazil**  (UM) (AM) | **Brazil**  (UM) (AM) | **India**  (LM) (SEA**)** |  | **India**  (LM) (SEA) | **India**  (LM) (SEA) | **India**  (LM) (SEA) | **India**  (LM) (SEA) | **Brazil**  (UM) (AM) | **Ethiopia**  (L) (AF) | **Philippines**  (LM) (WP) | **Malaysia**  (UM) (WP) |  | **Philippines**  (LM) (WP) | **India**  (LM) (SEA) | **India**  (LM) (SEA) |
| **Bangladesh**  (LM) (SEA) | **Bangladesh**  (LM) (SEA) | **Zambia**  (LM) (AF) | **Ethiopia**  (L) (AF) |  | **Ethiopia**  (L) (AF) | **Brazil**  (UM) (AM) | **Brazil**  (UM) (AM) | **Brazil**  (UM) (AM) | **Ethiopia**  (L) (AF) | **Palestine**  (UM) (EM) |  | **Nigeria**  (LM) (AF) |  |  | **Brazil**  (UM) (AM) | **Brazil**  (UM) (AM) |
| **Peru**  (UM) (AM) | **Peru**  (UM) (AM) | **Dominican Rep.**  (UM) (AM) | **Philippines**  (LM) (WP) |  | **South Africa**  (UM) (AF) | **Ethiopia**  (L) (AF) | **Ethiopia**  (L) (AF) | **Ethiopia**  (L) (AF) | **South Africa**  (UM) (AF) |  |  |  |  |  | **Ethiopia**  (L) (AF) | **Ethiopia**  (L) (AF) |
| **Malaysia**  (UM) (WP) | **Philippines**  (LM) (WP) | **Philippines**  (LM) (WP) | **Indonesia**  (UM) (SEA) |  | **Bangladesh**  (LM) (SEA**)** | **South Africa**  (UM) (AF) | **South Africa**  (UM) (AF) | **South Africa**  (UM) (AF) | **Peru**  (UM) (AM) |  |  |  |  |  | **Malaysia**  (UM) (WP) | **South Africa**  (UM) (AF) |
| **Pakistan**  (LM) (EM) |  | **Central Af. Rep.**  (L) (AF) |  |  | **Dominican Rep.**  (UM) (AM) | **Bangladesh**  (LM) (SEA) | **Bangladesh**  (LM) (SEA) | **Bangladesh**  (LM) (SEA) | **Chad**  (L) (AF) |  |  |  |  |  | **Chad**  (L) (AF) | **Bangladesh**  (LM) (SEA) |
|  |  |  |  |  | **Botswana**  (UM) (AF) | **Eswatini**  (LM) (AF) | **Dominican Rep.**  (UM) (AM) | **Dominican Rep.**  (UM) (AM) | **Mozambique**  (L) (AF) |  |  |  |  |  | **Mozambique**  (L) (AF) | **Eswatini**  (LM) (AF) |
|  |  |  |  |  | **Philippines**  (LM) (WP) | **Dominican Rep.**  (UM) (AM) | **Botswana**  (UM) (AF) | **Botswana**  (UM) (AF) | **China**  (UM) (WP) |  |  |  |  |  | **Pakistan**  (LM) (EM) | **Botswana**  (UM) (AF) |
|  |  |  |  |  | **Malawi**  (L) (AF) | **Peru**  (UM) (AM) | **Philippines**  (LM) (WP) | **Peru**  (UM) (AM) | **Nigeria**  (LM) (AF) |  |  |  |  |  | **China**  (UM) (WP) | **Philippines**  (LM) (WP) |
|  |  |  |  |  | **Indonesia**  (UM) (SEA) | **Philippines**  (LM) (WP) | **Malawi**  (L) (AF) | **Philippines**  (LM) (WP) | **Bolivia**  (LM) (AM) |  |  |  |  |  | **Nigeria**  (LM) (AF) | **Malaysia**  (UM) (WP) |
|  |  |  |  |  |  | **Malawi**  (L) (AF) | **Mozambique**  (L) (AF) | **Malaysia**  (UM) (WP) | **Palestine**  (UM) (EM) |  |  |  |  |  | **Palestine**  (UM) (EM) | **Chad**  (L) (AF) |
|  |  |  |  |  |  | **Guatemala**  (UP) (AM) | **Guatemala**  (UP) (AM) | **Malawi**  (L) (AF) | **Indonesia**  (UM) (SEA) |  |  |  |  |  | **Indonesia**  (UM) (SEA) | **Malawi**  (L) (AF) |
|  |  |  |  |  |  | **Central Af. Rep.**  (L) (AF) | **Pakistan**  (LM) (EM) | **Mozambique**  (L) (AF) |  |  |  |  |  |  |  | **Mozambique**  (L) (AF) |
|  |  |  |  |  |  | **Bolivia**  (LM) (AM) | **Colombia**  (UM) (AM) | **Pakistan**  (LM) (EM) |  |  |  |  |  |  |  | **Pakistan**  (LM) (EM) |
|  |  |  |  |  |  | **Palestine**  (UM) (EM) | **Bolivia**  (LM) (AM) | **Guatemala**  (UP) (AM) |  |  |  |  |  |  |  | **Guatemala**  (UP) (AM) |
|  |  |  |  |  |  | **Indonesia**  (UM) (SEA) | **Palestine**  (UM) (EM) | **China**  (UM) (WP) |  |  |  |  |  |  |  | **China**  (UM) (WP**)** |
|  |  |  |  |  |  |  | **Indonesia**  (UM) (SEA) | **Indonesia**  (UM) (SEA**)** |  |  |  |  |  |  |  | **Indonesia**  (UM) (SEA) |
|  |  |  |  |  |  |  |  | **Nigeria**  (LM) (AF) |  |  |  |  |  |  |  | **Nigeria**  (LM) (AF) |
|  |  |  |  |  |  |  |  | **Bolivia**  (LM) (AM) |  |  |  |  |  |  |  | **Palestine**  (UM) (EM) |

**Notes:**

- Article headings are based on *A/INB/9/3,Revised draft of the negotiating text of the WHO Pandemic Agreement, 13 March 2024* (<https://apps.who.int/gb/inb/pdf_files/inb9/A_inb9_3-en.pdf>).
- Changes to the Article headings from previous A/INB/7/3 (Proposal for negotiating text of the WHO Pandemic Agreement, 30 October 2023) are in *italicised* *and underlined* text.
- Income group is per World Bank Country and Lending Groups (2024 fiscal year) (<https://datahelpdesk.worldbank.org/knowledgebase/articles/906519-world-bank-country-and-lending-groups>).
  - **Upper-middle income = (UM); Lower-middle income = (LM); Low income = (L).**
- WHO Regions: Africa Region **(AF)**; Region of the Americas **(AM)**; Eastern Mediterranean Region **(EM)**; European Region **(EUR)**; South-East Asia Region **(SEA)**; and Western Pacific Region **(WP)** (<https://www.who.int/countries/>).
- A Member State is only counted once under each Article, even if they made more than one reference to the Article during their intervention
- India delivered the statement on behalf of South-East Asia.
- Ethiopia delivered a statement on behalf of the 47 Member States of the African Region and the Arab Republic of Egypt.
- Bangladesh delivered a statement on behalf of the members of the Group for Equity; Indonesia delivered a statement on behalf of the Group for Equity (Closing session).
